# Incorporating Known Genetic Variants Does Not Improve the Accuracy of PSA Testing to Identify High Risk Prostate Cancer on Biopsy

**DOI:** 10.1371/journal.pone.0136735

**Published:** 2015-10-02

**Authors:** Rebecca Gilbert, Richard M. Martin, David M. Evans, Kate Tilling, George Davey Smith, John P. Kemp, J. Athene Lane, Freddie C. Hamdy, David E. Neal, Jenny L. Donovan, Chris Metcalfe

**Affiliations:** 1 School of Social and Community Medicine, University of Bristol, Bristol, United Kingdom; 2 MRC Integrative Epidemiology Unit, University of Bristol, Bristol, United Kingdom; 3 University of Queensland Diamantina Institute, Translational Research Institute, Brisbane, Queensland, Australia; 4 Nuffield Department of Surgical Sciences, University of Oxford, Oxford, United Kingdom; 5 Department of Oncology, University of Cambridge, Cambridge, United Kingdom; Carolina Urologic Research Center, UNITED STATES

## Abstract

**Introduction:**

Prostate-specific antigen (PSA) testing is a widely accepted screening method for prostate cancer, but with low specificity at thresholds giving good sensitivity. Previous research identified four single nucleotide polymorphisms (SNPs) principally associated with circulating PSA levels rather than with prostate cancer risk (*TERT rs2736098*, *FGFR2 rs10788160*, *TBX3 rs11067228*, *KLK3 rs17632542*). Removing the genetic contribution to PSA levels may improve the ability of the remaining biologically-determined variation in PSA to discriminate between high and low risk of progression within men with identified prostate cancer. We investigate whether incorporating information on the PSA-SNPs improves the discrimination achieved by a single PSA threshold in men with raised PSA levels.

**Materials and Methods:**

Men with PSA between 3-10ng/mL and histologically-confirmed prostate cancer were categorised as high or low risk of progression (Low risk: Gleason score≤6 and stage T1-T2a; High risk: Gleason score 7–10 or stage T2C). We used the combined genetic effect of the four PSA-SNPs to calculate a genetically corrected PSA risk score. We calculated the Area under the Curve (AUC) to determine how well genetically corrected PSA risk scores distinguished men at high risk of progression from low risk men.

**Results:**

The analysis includes 868 men with prostate cancer (Low risk: 684 (78.8%); High risk: 184 (21.2%)). Receiver operating characteristic (ROC) curves indicate that including the 4 PSA-SNPs does not improve the performance of measured PSA as a screening tool for high/low risk prostate cancer (measured PSA level AU C = 59.5% (95% CI: 54.7,64.2) vs additionally including information from the 4 PSA-SNPs AUC = 59.8% (95% CI: 55.2,64.5) (p-value = 0.40)).

**Conclusion:**

We demonstrate that genetically correcting PSA for the combined genetic effect of four PSA-SNPs, did not improve discrimination between high and low risk prostate cancer in men with raised PSA levels (3-10ng/mL). Replication and gaining more accurate estimates of the effects of the 4 PSA-SNPs and additional variants associated with PSA levels and not prostate cancer could be obtained from subsequent GWAS from larger prospective studies.

## Introduction

Prostate cancer is the second most commonly diagnosed cancer and the 5th most common cause of cancer death worldwide for males [1]. Prostate-specific antigen (PSA) testing, followed by a prostate biopsy if the PSA level is raised (typically PSA 3-4ng/mL), is a widely accepted screening method for the disease. Early diagnosis and treatment may impact on survival in some men, but the majority of screen-detected prostate cancers are at low risk of progression, with potential harm caused by unnecessary diagnosis and treatment [2,3].

Despite the widespread use of PSA testing for prostate cancer, it has limited sensitivity and specificity. Men with a raised PSA may have no evidence of prostate cancer at biopsy, whilst not all men with prostate cancer have raised PSA: a third of men with PSA>10ng/mL showed no evidence of prostate cancer at sextant biopsy [4] whilst 63% of men with prostate cancer, and 41% of men with intermediate to high grade cancer (Gleason score≥7), had PSA<3ng/mL [5]. Results from the Prostate Cancer Prevention Trial found that a PSA threshold of 1.1ng/mL was required to achieve a sensitivity of 83.4%, but at the price of a false-positive rate of 61.1% (i.e. specificity = 39.9%)[6]. Among men with Gleason grade ≥7 (versus men with Gleason grade <7 or no cancer), a PSA threshold of 3.1ng/mL gave a sensitivity of 57.6% and specificity of 82.3%. Improving the interpretation of measured PSA levels may improve the clinical utility of the test, saving some men invasive unnecessary biopsies whilst ensuring that men with high risk prostate cancers are identified and offered treatment appropriately.

One potential approach to improve the sensitivity and specificity of PSA testing when identifiying prostate cancers at high risk of progression from those at low risk is to incorporate information on genetic variants. Inherited factors are thought to account for 40–45% of the variability in PSA levels, although these factors are largely unknown [7,8]. Gudmundsson [9] identified four SNPs that were principally associated with PSA levels rather than with prostate cancer risk in a genome-wide association study (GWAS) on serum PSA levels in Icelandic men not diagnosed with prostate cancer (PSA-SNPs: *TERT rs2736098*, *FGFR2 rs10788160*, *TBX3 rs11067228*, *KLK3 rs17632542*). Men who carried a high number of the PSA-SNP alleles that increase PSA levels were considered to be genetically “high” PSA producers, while those who carried decreased numbers were genetically “lower” PSA producers. They suggested that using the combined effect of these PSA-SNPs to genetically correct measured PSA might improve the performance of PSA as a screening tool for high risk prostate cancer.

The current paper aims to investigate whether the four PSA-SNPs can improve the sensitivity and specificity of PSA testing when identifying prostate cancer at high versus low risk of progression in men with a raised PSA level. We focus on men with a raised PSA level between 3-10ng/mL, where specificity is low and whether or not biopsy can be avoided in these men is clinically uncertain. We included men with PSA 3-10ng/mL who had histologically-confirmed prostate cancer after receiving a biopsy from a large UK-wide population-based case-control study (nested within the case-finding phase of the Prostate testing for cancer and Treatment (ProtecT) randomised controlled trial)[10,11]. Firstly, we tested whether the previously reported associations between PSA-SNPs and PSA levels, identified in men who were not diagnosed with prostate cancer, would be evident in our men who are diagnosed with prostate cancer. Secondly, we hypothesised that men with genetically “high” PSA are less likely to have high risk (vs low risk) prostate cancer as their high PSA is not entirely due to the presence of prostate cancer. Thirdly, we use the combined genetic effect of the four PSA-SNPs to genetically correct PSA level, and hypothesise that this corrected level would better identify prostate cancers at high risk of progression compared to those with low risk than the standard single PSA threshold (PSA≥3ng/mL). Fourthly, we investigated whether a greater improvement in identifying high (vs low) risk prostate cancers could be achieved by including both genetic correction of PSA levels and the combined effect of risk variants thought to be associated with aggressive (vs indolent) prostate cancer.

## Materials and Methods

### Participants

The study is nested within a multi-centre randomized controlled trial of treatments for localized prostate cancer: the Prostate Testing for cancer and Treatment (ProtecT) study (UK National Institute for Health Research (NIHR) Health Technology Assessment (HTA) Programme; ISRCTN 20141297)[10–12]. During recruitment to ProtecT between 2001 and 2009, over 100,000 men aged 50–69 years at 337 general practices in nine UK centres (Birmingham, Bristol, Cambridge, Cardiff, Edinburgh, Leeds, Leicester, Newcastle, Sheffield) were offered a PSA test at a community-based ‘prostate check clinic’, and those with raised levels (≥ 3 ng/mL) were offered diagnostic biopsy. Detected tumours were histologically-confirmed, clinically staged (“localized”: T1-T2; “locally advanced”: T3-T4)[13], and Gleason graded.

Men were included in the current study if they had a raised PSA (between ≥3ng/mL and <10ng/mL), available information on the relevant SNPs and a positive biopsy result with recorded stage or Gleason score. Men with stage T3-T4 were excluded, as stage is detected clinically and these men would be sent for biopsy regardless of any genetic correction. Men were categorised as high or low risk of progression according to their Gleason score and stage (Low risk: Gleason score ≤6 and stage T1-T2a; High risk: Gleason score 7–10 or stage T2C. If men were missing stage, we used Gleason score only). Only subjects who self-identified as white were included (99% of ProtecT cohort). The flow of participants is shown in S1 Fig.

### Genotyping in ProtecT

SNPs relevant to the current analysis were obtained from genome-wide genotyping of ProtecT, carried out on 3,390 individuals at the Centre National de Génotypage, Evry, France, using the Illumina Human660W-Quad_v1_A arrays (Illumina, Inc., San Diego, CA)[14]. The quality control process done before imputation excluded individuals on the basis of the following: sex mismatches, minimal (<0.325) or excessive heterozygosity (>0.345), disproportionate levels of individual missingness (>3%), cryptic relatedness measured as proportion of identity by descent (IBD > 0.1). The remaining individuals were assessed for evidence of population stratification by multidimensional scaling analysis and compared with HapMap II (release 22) European descent (CEU), Han Chinese (CHB), Japanese (JPT), and Yoruba (YRI) reference populations; all individuals with non-European ancestry were removed. SNPs with a minor allele frequency below 1%, a call rate of <95% or evidence for violations of Hardy—Weinberg equilibrium (P < 5 * 10^−7^) were discarded. Autosomal genotypic data were subsequently imputed using Markov Chain Haplotyping software (MACH v.1.0.16 [15]) and phased haplotype data from CEU individuals (HapMap release 22, Phase II NCBI B36,dbSNP 126) based on a cleaned dataset of 3,186 individuals and 514,432 autosomal SNPs. After imputation, all SNPs with indication of poor imputation quality (r^2^ hat < 0.30) were removed. X chromosome imputation was done on a cleaned dataset of 3,186 individuals and 10,092 X chromosome SNPs, using MACH v.1.0.16 and MiniMac v 4.4.3, in conjunction with phased haplotype data from CEU individuals (HapMap 3 release 2, NCBI B36, dbSNP 126). Genotypes were checked for deviation from Hardy-Weinberg equilibrium using the hwsnp function implemented in Stata (Stata Corporation, College Station, Texas).

Each SNP genotype was coded as 0, 1 or 2 depending on the number of risk alleles the individual carries. Information on the four SNPs principally associated with PSA levels rather than with prostate cancer risk (PSA-SNPs: *TERT* on 5p15.33 *rs2736098*, *FGFR2* on 10q26.12 *rs10788160*, *TBX3* on 12q24.21 *rs11067228*, *KLK3* on 19q13.33 *rs17632542*) was linked to the study characteristics of the included subjects.

#### Estimating Genetically Corrected PSA risk scores

We calculated the combined genetic effect by multiplying together the relative effect sizes based on published coefficients of each of the four PSA-SNPs from an Icelandic discovery population [9], each included to the power 0,1 or 2 depending on the number of risk alleles (0,1,2) of each SNP carried by an individual. No man in our data had 0 risk alleles for the SNP *rs17632542*. To account for this when calculating the combined genetic effect, the SNP *rs17632542* was included to the power 0 if a man had 1 copy of the risk allele and to the power 1 if the man had 2 copies. The increase in PSA level per allele (%) was determined from a previous GWAS based on Icelandic data [9]: *rs2736098*-A 10.5%; *rs10788160*-A 10.2%; *rs11067228*-A 8.3%; *rs17632542*-T 39.1%; giving relative allelic effects of 1.11, 1.10, 1.08, and 1.39 respectively. We attempted to confirm these relative allelic effects in the current data as part of our analysis. A genetically corrected PSA risk score, including information on the 4 PSA-SNPs, was estimated as measured PSA minus the combined genetic effect of the 4 PSA-SNPs where a higher score indicates that a man has a PSA that is greater than would be predicted by his genotypes. In other words,

Genetically corrected PSA risk score = PSA–(*rs2736098* ^i^ * *rs10788160* ^j^ * *rs11067228* ^k^ * *rs17632542* ^l^)

where i,j,k = 0,1,2 and l = 0,1 depending on the number of risk alleles (0,1,2) of each SNP carried by an individual for SNPs *rs2736098* (i), *rs10788160* (j), *rs11067228* (k) and *rs17632542* (l) respectively.

#### Including the combined effect of prostate cancer risk variants

We investigated whether additionally including the effect of 10 SNPs found in previous GWAS to be associated with aggressive prostate cancer could improve our ability to distinguish between high and low risk prostate cancer. The 10 SNPs and their associated effects are: *ATP5SL/CEACAM21 rs11672691*: OR per G allele increase = 1.12 (aggressive cases vs controls)[16]; *TNRC6 rs11704416* OR per G allele = 0.94 (aggressive vs controls)[16]; *17p12 rs4054823* OR per T allele = 1.13 (aggressive vs non-aggressive)[17]; *10q21*.*12 rs10788165*: OR for TT vs GG/GT = 1.34 (aggressive vs controls)[18]; *10q21*.*12 rs10749408* OR for TT vs CT/CC = 1.26 (aggressive vs controls)[18]; *10q21*.*12 rs11199874* OR for GG vs AG/AA = 1.42 (aggressive vs controls)[18]; *15q21*.*1 rs4775302* OR for AG/AA vs GG = 1.41(aggressive vs controls)[18]; *15q21*.*1 rs1994198* OR for CT/TT vs CC = 1.34 (aggressive vs controls)[18]; *DAB2IP rs1571801* OR for AC/CC vs AA = 1.36 (aggressive vs controls)[19]; *HERC2 rs6497287* OR for TC/CC vs TT = 1.46 (aggressive vs controls) [20]. The combined genetic effect was calculated by multiplying together the relative effect sizes of each of the five SNPs based on published coefficients depending on the number of risk alleles (0,1,2) of each SNP carried by an individual.

#### Population Stratification

The top 10 principal components (PCs) that reflect the population’s genetic structure were estimated according to Price et al [21] from the genome-wide SNPs genotyped and cleaned as described above. All 10 PCs were included as covariates in regression models to account for confounding by population stratification where appropriate.

### Statistical Analysis

#### PSA SNPs, PSA level and Prostate Cancer Risk

The previously published associations of PSA SNPs with PSA level in men without prostate cancer were investigated using linear regression to examine the association of PSA level with individual SNPs within men with raised PSA, calculating a per allele effect overall and stratified by high or low risk of progression, adjusted for age, study centre and population stratification. The proportion of trait variability (R-squared) and the F statistic were calculated from unadjusted linear regression models as an indication of how much of the variability in PSA level is explained by each SNP.

Whether men with “genetically” high PSA, based on published coefficients [9], were more likely to have low (vs high) risk prostate cancer was investigated using logistic regression, controlling for age, study centre and population stratification, to estimate odds ratios (OR) and 95% confidence intervals (CI) quantifying the associations of SNPs with prostate cancer (high vs low risk). SNPs were included as single variants and effects were estimated per change in allele.

#### Assessing Genetically Corrected PSA risk scores

We used receiver operating characteristic (ROC) curves and calculated the area under the curve (AUC) to assess the ability of genetically corrected PSA risk scores to discriminate between high and low risk prostate cancer when compared to measured PSA.

#### Including the combined effect of prostate cancer risk variants

We estimated the posterior odds of a man having high risk prostate cancer as being the prior odds of a man having prostate cancer given his measured PSA level and age, multiplied by the likelihood ratio (LR) for the genetically corrected PSA risk score, calculated as sensitivity/(1-specificity). The likelihood ratio was used to determine whether the addition of SNPs usefully changes the probability that a man has high (vs low) risk prostate cancer. A likelihood ratio close to one indicates that incorporating genetic variants does not improve on the pre-test probabilities of having high (vs low) risk prostate cancer. We calculated four likelihood ratios for: (i) measured PSA; (ii) 4 PSA-SNPs based on published coefficients [9]; (iii) 10 aggressive prostate cancer SNPs based on published coefficients; and (iv) both (ii) and (iii). Sensitivity was fixed at 90% and the corresponding specificity was estimated from the ROC curves.

#### Sensitivity Analyses

Sensitivity analyses were carried out looking at the effect of (i) stratifying by age (<65 years, ≥65 years); (ii) including extra SNPs found to be less strongly associated with PSA level in the same GWAS from which the 4 PSA-SNPs were identified. We looked at including the effect of the 4 PSA-SNPs individually, instead of the combined effect of all 4 PSA-SNPs. To investigate the impact of using effect estimates calculated internally rather than using the published coefficients, we fitted four logistic regression models with high/low risk as the outcome and calculated the AUC of each model: Model 1: measured PSA only; model 2: measured PSA and 4 PSA-SNPs; model 3: measured PSA and 5 aggressive prostate cancer SNPs; and model 4: measured PSA, 4 PSA-SNPs and 5 aggressive prostate cancer SNPs. We repeated the analysis comparing very high grade (≥8) versus very low grade (5–6), since grade 7 is a mixture of more aggressive (Gleason score 4+3) and less aggressive (Gleason score 3+4) cancers. We also repeated the analysis additionally including men who were staged as T3-T4 in the high risk group.

Analyses were carried out in Stata 13 (StataCorp, 2013. College Station, TX). All tests of statistical significance were two-sided. All men provided written informed consent prior to inclusion in the study. Trent Multicentre Research Ethics Committee (MREC) approved the ProtecT study (MREC/01/4/025) and the associated ProMPT study which collected biological material (MREC/01/4/061).

## Results

### Characteristics of Men included

The analysis includes 868 men with raised PSA (≥3ng/mL and <10ng/mL) and histologically-confirmed prostate cancer that had information available on the 4 PSA-SNPs (828 (95.4%) localized (T1: 637, T2: 191), 40 (4.6%) missing stage; 684 (78.8%) low-grade, 184 (21.2%) high-grade; Low risk: 684 (78.8%); High risk: 184 (21.2%)).

The mean age of high risk men was 63.1 years and of low risk men was 62.4 years (p-for-difference = 0.08). Mean measured PSA was 4.9ng/mL (SD 1.7). The mean PSA level in men with high risk cancer was higher than low risk (5.4ng/mL vs 4.8ng/mL, p<0.001). There were no substantial differences in other baseline characteristics (S1 Table).

### PSA SNPs, PSA levels and Prostate Cancer Risk

There was no convincing evidence that associations of four PSA-SNPs with PSA level, previously reported in men without prostate cancer, were replicated in men with raised PSA (3-10ng/nL) and prostate cancer: none of the four PSA-SNPs were associated with PSA level in men stratified by cancer risk (high or low risk of progression) (Table 1). Approximately 2.4% of the variability in PSA levels within men at high risk of progression is explained by including all four PSA-SNPs simultaneously, and 0.9% within men at low risk of progression.

**Table 1:** The effects of SNPs on PSA level (ng/mL) in men with PSA3-10ng/mL and a diagnosis of prostate cancer.

				Low Risk (N = 684)					High (N = 226)			
SNP	Alleles (O/X)	Allele associated with Increasing PSA (X)	Effect of X allele on PSA (ng/mL)^a^	95% CI	p-value	F- statistic^b^	R^2^ (%)	Effect of X allele on PSA (ng/mL)^a^	95% CI	p-value	F- statistic^b^	R^2^ (%)
rs10788160	G/A	A	-0.16	(-0.36, 0.03)	0.10	3.08	0.45	-0.28	(-0.76, 0.20)	0.26	0.54	0.30
rs11067228	G/A	A	-0.13	(-0.30, 0.04)	0.14	1.95	0.29	-0.38	(-0.81, 0.05)	0.08	3.18	1.72
rs17632542	C/T	T	0.17	(-0.21, 0.56)	0.38	0.65	0.09	0.52	(-0.29, 1.34)	0.21	0.77	0.42
rs2736098	C/T	T	0.05	(-0.14, 0.25)	0.60	0.19	0.03	0.14	(-0.34, 0.61)	0.57	0.43	0.24

CI = confidence interval.

^a^ Additive model, calculated using regression, adjusting for exact age, study centre and 10 principal components to account for confounding by population stratification.

^b^ F-statistic and R-squared (R^2^) indicate how much of the variability in PSA levels is explained by each SNP. Calculated using regression, unadjusted

For *rs17632542*, there was some evidence that men with “genetically” high PSA, i.e. had 1 or 2 copies of the T-allele associated with increasing PSA, had a decreased risk of prostate cancer at high risk of progression (*rs17632542*-T: OR per allele 0.62, CI: 0.38,1.00). There was no convincing evidence that the other three SNPs were associated with prostate cancer at high risk of progression compared to low risk (Table 2). There was no evidence that the ten aggressive prostate cancer SNPs were associated with prostate cancer at high risk of progression compared to low risk (Table 2).

**Table 2:** Associations between SNPs and prostate cancer risk (high versus low risk) in men with PSA3-10ng/mL.

		N (high vs low risk)			Effect of X allele^a^		
SNP	Alleles (O/X)	OO	OX	XX	OR	(95% CI)	p-value
**4 PSA-SNPs**							
rs10788160	G/A	107/391	68/249	9/44	0.93	(0.70, 1.22)	0.59
rs11067228	G/A	25/131	95/332	64/221	1.21	(0.95, 1.55)	0.12
rs17632542	C/T	./.	29/75	155/609	0.62	(0.38, 1.00)	0.05
rs2736098	C/T	80/280	91/350	13/54	0.93	(0.70, 1.22)	0.59
**10 Aggressive Prostate Cancer SNPs**							
rs10749408	C/T	79/324	86/278	19/82	1.03	(0.81, 1.32)	0.79
rs10788165	G/T	83/293	88/312	13/79	0.85	(0.66, 1.10)	0.21
rs11199874	A/G	106/391	69/249	9/44	0.94	(0.71, 1.24)	0.67
rs11672691	A/G	109/408	65/241	10/35	1.05	(0.79, 1.39)	0.76
rs11704416	C/G	107/446	69/211	8/27	1.25	(0.94, 1.67)	0.13
rs1571801	T/G	13/51	75/294	96/339	1.08	(0.83, 1.41)	0.58
rs1994198	C/T	53/230	86/320	45/134	1.23	(0.97, 1.54)	0.08
rs4054823	C/T	63/224	86/321	35/139	0.93	(0.73, 1.18)	0.54
rs4775302	G/A	49/138	85/339	50/207	0.82	(0.65, 1.04)	0.1
rs6497287	C/T	158/578	25/101	1/5	0.9	(0.58, 1.40)	0.64

OR = odds ratio; CI = confidence interval; p = p-value. The X allele would be expected to be associated with increased risk.

^a^ High vs low risk models are calculated using regression, adjusting for exact age, study centre and 10 principal components to account for confounding by population stratification. Additive model.

### Genetically Corrected PSA Risk Score

**Fig 1:**
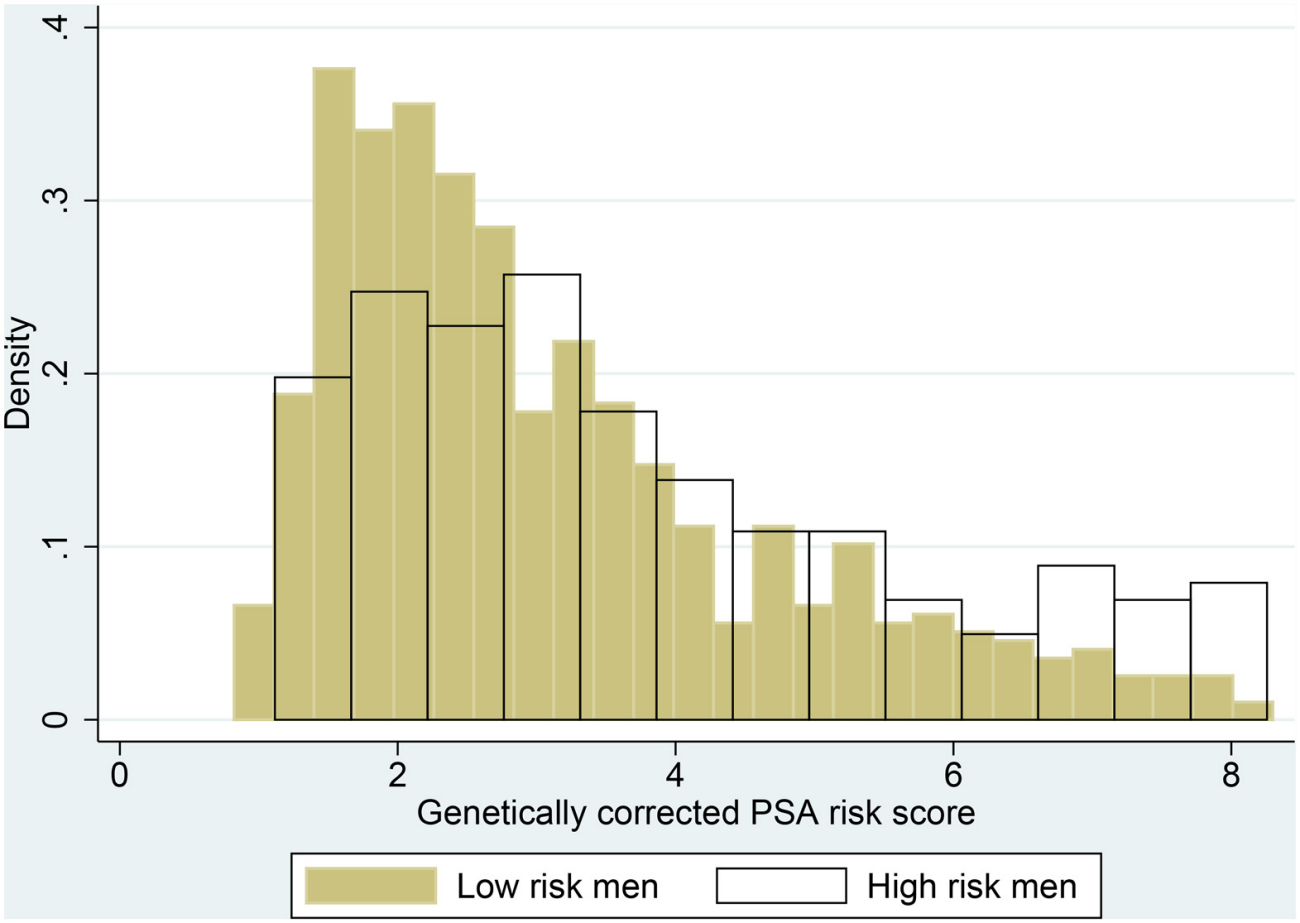
Histograms showing the distribution of genetically corrected PSA risk score. Histograms showing genetically corrected PSA risk score, including information on the 4 PSA-SNPs using the published coefficients, stratified by high versus low risk of progression show no difference between men at high and low risk of progression.

The median number of “high PSA” alleles across the 4 PSA-SNPs was 4 (range: 1–8). The mean PSA in men with 4 alleles was 4.9ng/mL (Table 3). The mean and range of the genetically corrected PSA risk score was 3.2 (0.8,8.3), where a higher score indicates that a man has a PSA that is greater than would be predicted by his genotypes. Histograms comparing genetically corrected PSA risk score using the published coefficients show no difference between men at high and low risk of progression (Fig 1).

**Table 3:** The mean PSA level (ng/mL) by the number of alleles across the 4 PSA-SNPs.

Number of alleles (1–8)	N	Mean (SD) PSA level (ng/mL)	
1	4	4.57	(1.82)
2	55	3.55	(1.38)
3	203	3.60	(1.83)
4	253	3.28	(1.68)
5	249	3.09	(1.64)
6	82	2.75	(1.60)
7	21	2.34	(1.52)
8	1	2.08	(0.00)

**Fig 2:**
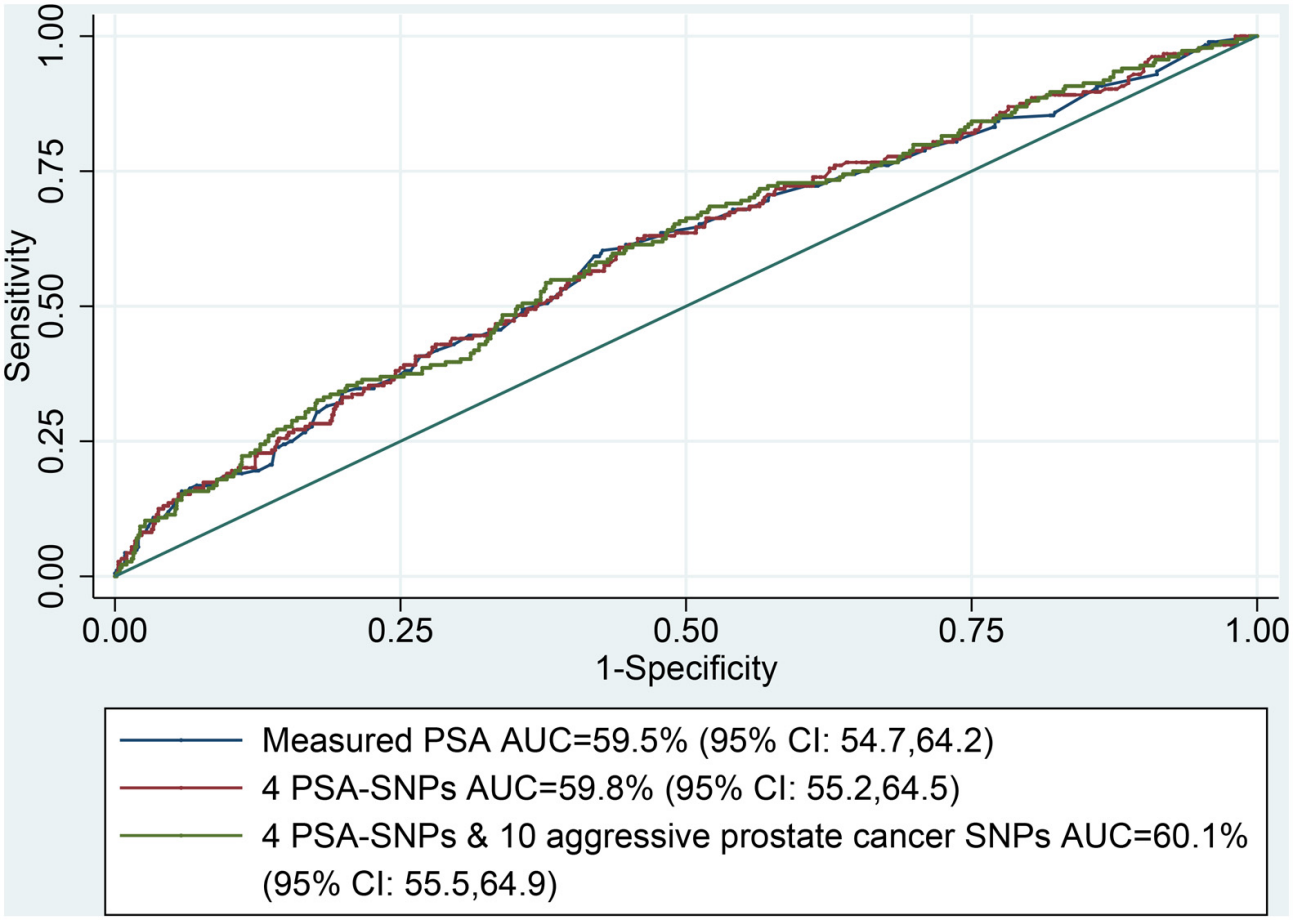
ROC curves comparing measured PSA to genetically corrected PSA risk score using the 4 PSA-SNPs. Area under the curve (AUC) comparing measured PSA, genetically corrected PSA risk score based on 4 PSA-SNPs using the published coefficients and PSA risk score corrected for both 4 PSA-SNPs and 10 aggressive prostate cancer SNPs using published coefficients.

ROC curves indicate that including the 4 PSA-SNPs using published coefficients does not improve the performance of measured PSA as a tool for identifying high risk prostate cancer (measured PSA level AUC = 59.5% (95% CI: 54.7,64.2) vs genetically corrected PSA risk scores using the 4 PSA-SNPs AUC = 59.8% (95% CI: 55.2,64.5) (p-value = 0.40)) (Fig 2). For a sensitivity of 90%, the ROC curve estimates the corresponding specificity to be 0.14 for measured PSA and 0.13 for genetically corrected PSA risk scores using 4 PSA-SNPs (p-for-difference = 0.83). This is equivalent to having offered biopsy to all men with a measured PSA of 3.3ng/mL (sensitivity = 90.2%, specificity = 14.2%).

### Including the combined effect of prostate cancer risk variants

ROC curves indicate that additionally including the 10 PSA-SNPs did not improve the performance of measured PSA as a tool for identifying high risk prostate cancer (measured PSA level AUC = 59.5% (95% CI: 54.7,64.2) vs additionally including the 4 PSA-SNPs and 10 aggressive prostate cancer SNPs AUC = 60.1% (95% CI: 55.5,64.9) (p-value = 0.43)) (Fig 2). For a sensitivity of 90%, the equivalent specificity and likelihood ratios were: (i) measured PSA: specificity = 14.2%, LR = 1.05; (ii) 4 PSA-SNPs: specificity = 13.3%, LR = 1.04; (iii) 10 aggressive prostate cancer SNPS: specificity = 5.8%, LR = 0.96; and (iv) both (ii) and (iii): specificity = 16.8%, LR = 1.08.

### Sensitivity Analyses

Stratifying the analysis by age (<65years, ≥65 years) did not improve the performance of measured PSA or genetically corrected PSA risk scores as a tool for identifying high risk prostate cancer (measured PSA level: AUC in men<65 years = 57.8% (95% CI: 51.7,63.9), AUC in men≥65 years = 61.2% (95% CI: 53.7,68.7), p-for-difference = 0.49; genetically corrected PSA risk score using 4 PSA-SNPs: in men<65 years = 58.0% (95% CI: 51.9,64.1), AUC in men≥65 years = 62.0% (95% CI: 54.6,69.4), p-for-difference = 0.41). Adding additional SNPs associated with PSA did not improve the model and so were not considered further (results not shown). Including the 4 PSA-SNPs individually did not improve performance (*rs2736098*: AUC = 59.6% (95% CI: 54.8,64.3); *rs10788160*: AUC = 59.4% (95% CI: 54.7,64.2); *rs11067228*: AUC = 59.3% (95% CI: 54.5,64.0); *rs17632542*: AUC = 59.9% (95% CI: 55.2,64.6)).

Using ROC curves to assess the discriminatory power of 4 models using internal effect estimates (as a sensitivity analysis rather than using the published coefficients) showed that additionally adding the effect of 4 PSA-SNPs (model 2), 10 aggressive prostate cancer SNPs (model 3) did not improve the performance of measured PSA alone (model 1) when identifying high risk prostate cancer from low risk: model 1 AUC = 59.5% (95% CI: 54.7,64.2), model 2 AUC = 62.0% (95% CI: 57.5,66.5), model 3 AUC = 61.7% (95% CI: 57.0,66.3). P-for-difference comparing model 2 with model 1 = 0.11 and model 3 with model 1 = 0.16). Additionally adding the combined effect of 4 PSA-SNPs and 10 aggressive prostate cancer SNPs (model 4) did slightly improve upon the performance of measured PSA alone (model 1) when identifying high risk prostate cancer from low risk: model 1 AUC = 59.5% (95% CI: 54.7,64.2), model 4 AUC = 63.6% (95% CI: 59.1,68.1). P-for-difference comparing model 4 with model 1 = 0.03).

Repeating the analysis comparing very high grade (≥8) (n = 23) versus very low grade (5–6) (n = 684) gave an AUC of 63.8% (CI: 52.8,74.8) for measured PSA level and an AUC of 65.1% (CI:54.5,75.7) for genetically corrected PSA risk score using 4 PSA-SNPs (p-for-difference = 0.27). Additionally adding the 10 prostate cancer SNPS gave an AUC of 63.4% (CI: 52.0,74.8). Repeating the analysis including men with locally advanced prostate cancer (clinically detected stage T3: n = 42, T4: n = 0) in the high risk group did not alter the results (results not shown).

### Population Stratification

There was no convincing evidence of an association between each SNP and score with the principal components used in this adjustment, indicating that population stratification was not likely to have affected our results (S2 Table).

## Discussion

### Summary of Findings

The genetic correction of each individual man’s PSA level did not improve discrimination between men at high and low risk of progression compared to the traditional single PSA threshold applied to all men when identifying prostate cancer at high risk of progression in men with a PSA level between 3-10ng/mL. Using a genetically corrected PSA risk score, determined from the combined genetic effect of the four PSA-SNPs, we identified 90% of high risk cancers whilst sparing 13% of low risk men an invasive test. The same results could be attained by increasing the single threshold from 3ng/mL to 3.3ng/mL. Adding information on 10 SNPs thought to be associated with aggressive (vs indolent) prostate cancer in previous studies did not improve our ability to distinguish high from low risk prostate cancer.

We found no convincing evidence that the 4 PSA-SNPs were associated with PSA levels in men with raised PSA (3-10ng/mL) who had histologically-confirmed prostate cancer, stratified by high and low risk of progression. There was little evidence that men with genetically”high” PSA, i.e. men who carry an increased number of the 4 PSA-SNP alleles, were more likely to have low risk (vs high risk) prostate cancer. Using internal effect estimates of the 4 PSA-SNPs did not improve on the single PSA threshold.

#### Previous Literature

Serum PSA elevation is a consequence rather than a cause of prostate cancer. PSA levels are also increased by age, urinary tract infections, and conditions such as benign prostatic hyperplasia and decreased by conditions such as obesity. Much work has focused on improving the predictive performance of the traditional single threshold PSA test using other risk factors for prostate cancer, for example PSA kinetics, age, ethnicity and family history of prostate cancer [22]. Previous studies have focused on identifying SNPs to predict the risk of prostate cancer, whereas the current aim is to use SNPs to inform PSA testing and identify prostate cancer at high risk of progression. Removing the genetic contribution to PSA levels may improve the ability of the remaining biologically determined variation in PSA to detect prostate cancer at high risk of progression.

Since 2006, 25 prostate cancer GWAS have been published and 76 susceptibility loci associated with prostate cancer risk have been identified [23]. The ability of SNPS to predict aggressive disease is unclear, with some [24–27], but not all [28], studies supporting an association between SNPs and aggressive prostate cancer. Control selection strategies, such as comparing cases to controls that have been selected on the basis of their PSA level being under a certain threshold, can make it difficult to interpret associations between a genetic variant and disease, since such associations may result from a relationship between the variant and PSA levels rather than prostate cancer [20,29–36]. A limited number of GWAS which used PSA screened controls found that thirteen known prostate cancer susceptibility loci are also associated with PSA concentration in blood (*rs6869841*, *rs1270884*, *rs17632542*, *rs2242652*, *rs6983561*, *rs620861*, *rs10090154*, *rs7837688*, *rs12500426*, *rs7127900*, *rs10993994*, *rs2659056*, *rs2735839*, *rs5945619*) [9,24,29,30,34].

Individual SNPs have been identified which explain a proportion of the variation in PSA level. Eeles et al [30] detected an association between a SNP that encodes PSA [*kallikrein-related peptidase 3 (KLK3)*] and prostate cancer. This SNP was subsequently associated with PSA levels in unaffected men, as were other prostate-cancer related SNPs in the *hepatocyte nucleartor-1 β (HNF1B)* and *β-microseminoprotein (MSMB)* genes [36–39]. Knipe et al [40] compared the association of 81 SNPs with prostate cancer using ‘low’ PSA controls and ‘high’ PSA controls, nested within ProtecT. They hypothesized that if the genetic marker was associated with PSA level and not prostate cancer, then the effect estimate would be greatest when using ‘low’ PSA controls and close to the null when using ‘high’ PSA controls. They found that seven SNPs were positively associated with circulating PSA level (*rs1512268-T*, *rs10788160-A*, *rs445114-T*, *rs11199874-A*, *rs17632542-T*, *rs2735839-G*, *rs266849-A*). Jin [41]conducted a GWAS of percentage of free-to-total PSA (%fPSA) by genotyping 642,584 SNPs in 3192 men of European ancestry, each with a total PSA of 2.5 to 10ng/mL, that were recruited to the REDUCE study. They identified two SNPs (*rs3213764*, *rs1354774*) that were associated with %fPSA but not associated with prostate cancer risk or aggressiveness. The SNP *rs3213764* was also associated with total PSA; rs1354774 was not associated with total PSA.

Only one study specifically investigated the relationship between genetic variants and circulating PSA level in men without detected prostate cancer. Gudmundsson’s [9] GWAS on serum PSA levels identified 4 PSA-SNPs that were principally associated with PSA levels rather than with prostate cancer risk. This GWAS was conducted in Icelandic men not diagnosed with prostate cancer according to data from the nationwide Icelandic Cancer Registry (ICR) (n = 15,757; mean (sd) age: 63 years (12); median (IQR) PSA level: 2 (0.8,4.4) and results were replicated in a subsample of men from the ProtecT trial with PSA<3ng/mL who had not undergone prostate biopsies (n = 454; mean (sd) age: 63 (5); median (IQR) PSA: 1.5 (0.7,2.2)). Despite finding no convincing evidence that the four PSA-SNPs were associated with PSA levels in the current data, two of our results are in the same direction as the association observed by Gudmundsson (rs2736098-T and rs17632542-T were associated with increased PSA). Using the combined effect of the four PSA-SNPs to genetically correct measured PSA, they found that 6–7% of Icelandic men undergoing PSA-based prostate cancer screening would have at least one PSA measurement reclassified with respect to whether they should undergo a biopsy (using a threshold for biopsy of PSA≥4ng/mL). However, using the combined effect of these PSA-SNPs to genetically correct measured PSA did not alter the performance of PSA as a screening tool for prostate cancer (PSA adjusted for the four PSA-SNPs AUC 70.9% compared to unadjusted PSA AUC 70.4% in 415 men who underwent biopsy, p-for-difference not given). Additionally adding the combined risk of 23 prostate cancer risk variants increased the AUC to 73.2%. In data from the ProtecT trial (n = 1291), unadjusted PSA had an AUC of 57.1%, PSA adjusted for 4 PSA-SNPs had an AUC 58.5% and the AUC when including the 23 prostate cancer risk variants was 63.3%.

Hefland [42] used the 4 PSA-SNPs to create personalized PSA cutoffs and to determine how many men would meet common biopsy criteria (PSA ≥2.5ng/mL and ≥4ng/mL) after genetic correction of their measured PSA level (n = 964 US Caucasian men without prostate cancer). They found that genetic correction lead to a 15–20% relative reduction in the number of biopsies received and a possible 18–22% reduction in the number of potentially unnecessary biopsies. However, not all participants had undergone biopsy so true confirmation of reclassification after genetic correction as potentially unnecessary biopsy remains to be determined prospectively.

A number of other studies have found that PSA testing may be improved by genetic correction including SNPs which are associated with both PSA level and prostate cancer risk [42–45]. Results from the Stockholm-1 cohort [45] found that the addition of a genetic risk score including 35 SNPs resulted in fewer biopsies than a non-genetic model (avoiding 22.7% of biopsies) at a cost of missing a prostate cancer diagnosis in 3% of patients with aggressive cancer (5241 men (2135 with prostate cancer) with PSA≤10ng/mL). Johansson [44] concluded that the addition of a genetic risk score made up of 33 common genetic variants to PSA resulted in a marginal improvement in prostate cancer prediction (520 cases, 988 controls, AUC for genetic risk score = 64.3%, AUC for PSA = 86.2%, AUC for PSA and genetic risk score = 87.2%). Two other studies found that the addition of SNPs made little difference in the ability of PSA to predict prostate cancer [35,46]. In the current data, the addition of 10 SNPs thought to be associated with aggressive prostate cancer did not improve our ability to distinguish between high and low risk prostate cancers beyond that of the four PSA-SNPs, in men with PSA levels between 3-10ng/mL.

Two of the 4 PSA-SNPs may not be specific to prostate cancer. *TERT* encodes an enzyme, telomerase, whose increased activity is associated with malignant tumors due to its role in proliferation and apoptosis of cancer cells [47] and has been found to be active in 90% of human cancers [48]. Similarly, fibroblast growth factors play a role in promoting proliferation and differentiation of cells, and mediating angiogenesis, and *FGFR2* has been consistently found to be associated with breast cancer [49]. The NHGRI GWAS catalog [50] (accessed on 17^th^ September 2014) identified 21 published GWAS linking *TERT* to breast cancer, bladder cancer, testicular cancer, lung cancer, glioma and prostate cancer and 11 GWAS linking *FGFR2* to breast cancer. This suggests that at least two of the 4 PSA-SNPs might not be specific to PSA level and may still be associated with prostate cancer after all. Our sensitivity analysis adjusting for the 4 PSA-SNPs individually, instead of the combined effect of all 4, showed that no single PSA-SNP improved our model.

#### Strengths and Limitations

We did not include men with PSA<3ng/mL: since the decision to biopsy was based on PSA level, some of the controls with PSA<3ng/mL will have unidentified prostate cancer [6] (misclassification bias [51]). This would not affect our analysis of men with PSA≥3ng/mL as all cancers were biopsy confirmed. We have included men with PSA between 3-10ng/ml, as whether or not biopsy can be avoided in these men is clinically uncertain and it is likely that men with PSA>10ng/mL would be biopsied regardless of any genetic correction.

By including all four PSA-SNPs in the risk score irrespective of whether they replicated in the current dataset or not, we avoid over-estimating the performance of our genetically corrected PSA risk score because of over-fitting to the current dataset. The estimates used to calculate PSA-SNP score were taken from one GWAS on PSA levels carried out in Icelandic men not diagnosed with prostate cancer (n = 15,757). Similar results were found in their follow-up analysis of 454 men with PSA<3ng/mL from the ProtecT study. It has been shown that results from the first study on a topic often suggest a stronger effect than is found in subsequent studies, and that genetic association studies require cautious replication [52]. Confirmation in other datasets is required to ensure that these 4 PSA-SNPs are truly associated with PSA and not prostate cancer. It is likely that the proportion of variance explained by the 4 PSA-SNPs is not large enough to meaningfully correct PSA. The effect of having prostate cancer on PSA level may be so large that it could mask the comparatively small genetic contribution. More accurate estimates of the effects of the 4 PSA-SNPs and additional variants associated with PSA levels and not prostate cancer could be obtained from subsequent GWAS from larger prospective studies. Most GWAS to date have compared population controls to cases mainly affected with low-risk disease, whereas we have attempted to identify high risk from low risk prostate cancers. Our results apply to screen-detected prostate cancers and may not be generalizable to clinically detected cancer. Screen-detecting cancers may have resulted in a narrow range of aggressiveness, making it harder to demonstrate an association. Grade was based on biopsy material, and could potentially be upgraded if the man underwent radical prostatectomy. It is possible that our results are affected by inaccurate grading and tumour heterogeneity insufficiently captured by biopsy. Neither adding further SNPs associated with PSA level, stratifying by age group (<65 years, ≥65 years) nor using internal effect estimates improved the model. The model was improved when discriminating between very high grade (≥8) and low grade (5–6) prostate cancers, but this analysis was based on only 23 very high grade cancers and so was underpowered. Moreover, adding SNPs associated with aggressive prostate cancer does not improve this model further.

## Conclusion

Our study of 868 men with raised PSA (3-10ng/mL) and histologically-confirmed prostate cancer has demonstrated that genetically correcting PSA for the combined genetic effect of four PSA-SNPs, did not improve upon the traditional single PSA threshold for biopsy when distinguishing high and low risk disease.

## Supporting Information

S1 Data De-identified Dataset. (XLS)

S1 Fig Flowchart of Participants. (DOC)

S1 Table Baseline characteristics of men with prostate cancer and PSA> = 3ng/mL and <10ng/mL included in the study. (DOC)

S2 Table The association between SNPs and principal components in men with prostate cancer and PSA 3-10ng/mL. (DOC)

## References

[1] Cancer Research UK (2014) CancerStats report Prostate Cancer—UK, Cancer Research UK. Available: http://infocancerresearchukorg/cancerstats/types/prostate.

[2] Schroder FH, Hugosson J, Roobol MJ, Tammela TLJ, Ciatto S, Nelen V, et al (2009) Screening and Prostate-Cancer Mortality in a Randomized European Study. The New England Journal of Medicine 360: 1320–1328. 10.1056/NEJMoa0810084 19297566

[3] Loeb S, Bjurlin MA, Nicholson J, Tammela TL, Penson DF, Carter HB, et al (2014) Overdiagnosis and Overtreatment of Prostate Cancer. European Urology 65: 1046–1055. 10.1016/j.eururo.2013.12.062 24439788 PMC4113338

[4] Aus G, Damber J, Khatami A, Lilja H, Stranne J, Hugosson J. (2005) Individualized screening interval for prostate cancer based on prostate-specific antigen level: Results of a prospective, randomized, population-based study. Archives of Internal Medicine 165: 1857–1861. 16157829 10.1001/archinte.165.16.1857 PMC1950470

[5] Thompson IM, Ankerst DP, Chi C, Goodman PJ, Tangen CM, Lucia MS, et al (2006) Assessing Prostate Cancer Risk: Results from the Prostate Cancer Prevention Trial. JNCI Journal of the National Cancer Institute 98: 529–534. 16622122 10.1093/jnci/djj131

[6] Thompson IM, Ankerst DP, Chi C, Lucia MS, Goodman PJ, Crowley JJ, et al (2005) Operating Characteristics of Prostate-Specific Antigen in Men With an Initial PSA Level of 3.0 ng/mL or Lower. JAMA: The Journal of the American Medical Association 294: 66–70. 15998892 10.1001/jama.294.1.66

[7] Bansal A, Murray DK, Wu JT, Stephenson RA, Middleton RG, Meikle AW. (2000) Heritability of Prostate-Specific Antigen and Relationship with Zonal Prostate Volumes in Aging Twins. Journal of Clinical Endocrinology & Metabolism 85: 1272–1276. 10720075 10.1210/jcem.85.3.6399

[8] Pilia G, Chen W-M, Scuteri A, Orrú M, Albai G, Dei M, et al (2006) Heritability of Cardiovascular and Personality Traits in 6,148 Sardinians. PLoS Genet 2: e132 16934002 10.1371/journal.pgen.0020132 PMC1557782

[9] Gudmundsson J, Besenbacher S, Sulem P, Gudbjartsson DF, Olafsson I, Arinbjarnarson S, et al (2010) Genetic Correction of PSA Values Using Sequence Variants Associated with PSA Levels. Science Translational Medicine 2: 62ra92 10.1126/scitranslmed.3001513 21160077 PMC3564581

[10] Lane JA, Donovan JL, Davis M, Walsh E, Down L, Turner EL, et al (2014) Active monitoring, radical prostatectomy, or radiotherapy for localised prostate cancer: study design and diagnostic and baseline results of the ProtecT randomised phase 3 trial. Lancet Oncol 15: 1109–1118. 10.1016/S1470-2045(14)70361-4 25163905

[11] Lane JA, Hamdy FC, Martin RM, Turner EL, Neal DE, Hamdy FC, et al (2010) Latest results from the UK trials evaluating prostate cancer screening and treatment: The CAP and ProtecT studies. European Journal of Cancer 46: 3095–3101. 21047592 10.1016/j.ejca.2010.09.016

[12] Gilbert R, Metcalfe C, Fraser WD, Donovan J, Hamdy FC, Neal DE, et al (2012) Associations of circulating 25-hydroxyvitamin D with prostate cancer diagnosis, stage and grade. International Journal of Cancer 131: 1187–1196. 22033893 10.1002/ijc.27327 PMC3378478

[13] Ohori M, Wheeler TM, Scardino PT (1994) The New American Joint Committee on Cancer and International Union Against Cancer TNM classification of prostate cancer. Clinicopathologic correlations. Cancer 74: 104–114. 7516262 10.1002/1097-0142(19940701)74:1<104::aid-cncr2820740119>3.0.co;2-5

[14] Bonilla C, Gilbert R, Kemp JP, Timpson NJ, Evans DM, Donovan J, et al (2013) Using genetic proxies for lifecourse sun exposure to assess the causal relationship of sun exposure with circulating vitamin D and prostate cancer risk. Cancer Epidemiology Biomarkers & Prevention 22: 597–606. 10.1158/1055-9965.EPI-12-1248 PMC3616836 23441100

[15] Li Y, Willer CJ, Ding J, Scheet P, Abecasis GR (2010) MaCH: Using Sequence and Genotype Data to Estimate Haplotypes and Unobserved Genotypes. Genetic Epidemiology 34: 816–834. 10.1002/gepi.20533 21058334 PMC3175618

[16] Amin Al Olama A, Kote-Jarai Z, Schumacher FR, Wiklund F, Berndt SI, Benlloch S, et al (2013) A meta-analysis of genome-wide association studies to identify prostate cancer susceptibility loci associated with aggressive and non-aggressive disease. Human Molecular Genetics 22: 408–415. 10.1093/hmg/dds425 23065704 PMC3526158

[17] Xu J, Zheng SL, Isaacs SD, Wiley KE, Wiklund F, Sun J, et al (2010) Inherited genetic variant predisposes to aggressive but not indolent prostate cancer. Proceedings of the National Academy of Sciences 107: 2136–2140. 10.1073/pnas.0914061107 PMC2836698 20080650

[18] Nam RK, Zhang W, Siminovitch K, Shlien A, Kattan MW, Klotz LH, et al (2011) New variants at 10q26 and 15q21 are associated with aggressive prostate cancer in a genome-wide association study from a prostate biopsy screening cohort. Cancer Biol Ther 12: 997–1004. 10.4161/cbt.12.11.18366 22130093 PMC3280918

[19] Duggan D, Zheng SL, Knowlton M, Benitez D, Dimitrov L, Wiklund F, et al (2007) Two genome-wide association studies of aggressive prostate cancer implicate putative prostate tumor suppressor gene DAB2IP. J Natl Cancer Inst 99: 1836–1844. 18073375 10.1093/jnci/djm250

[20] FitzGerald LM, Kwon EM, Conomos MP, Kolb S, Holt SK, Levine D et al (2011) Genome-wide association study identifies a genetic variant associated with risk for more aggressive prostate cancer. Cancer Epidemiol Biomarkers Prev 20: 1196–1203. 10.1158/1055-9965.EPI-10-1299 21467234 PMC3111761

[21] Price AL, Patterson NJ, Plenge RM, Weinblatt ME, Shadick NA, Reich D. (2006) Principal components analysis corrects for stratification in genome-wide association studies. Nat Genet 38: 904–909. 16862161 10.1038/ng1847

[22] Witte JS (2010) Personalized prostate cancer screening: improving PSA tests with genomic information. Science Translational Medicine 2: 62ps55 10.1126/scitranslmed.3001861 21160075

[23] Eeles R, Goh C, Castro E, Bancroft E, Guy M, Amin Al Olama A, et al (2014) The genetic epidemiology of prostate cancer and its clinical implications. Nat Rev Urol 11: 18–31. 10.1038/nrurol.2013.266 24296704

[24] Eeles RA, Al Olama AA, Benlloch S, Saunders EJ, Leongamornlert DA, Tymrakiewicz M, et al (2013) Identification of 23 new prostate cancer susceptibility loci using the iCOGS custom genotyping array. Nature genetics 45: 385–391. 10.1038/ng.2560 23535732 PMC3832790

[25] Cussenot O, Azzouzi AR, Bantsimba-Malanda G, Gaffory C, Mangin P, Cormier L, et al (2008) Effect of genetic variability within 8q24 on aggressiveness patterns at diagnosis and familial status of prostate cancer. Clin Cancer Res 14: 5635–5639. 10.1158/1078-0432.CCR-07-4999 18765558

[26] Helfand BT, Kan D, Modi P, Catalona WJ (2011) Prostate cancer risk alleles significantly improve disease detection and are associated with aggressive features in patients with a “normal” prostate specific antigen and digital rectal examination. The Prostate 71: 394–402. 10.1002/pros.21253 20860009 PMC3089434

[27] Cheng I, Plummer SJ, Neslund-Dudas C, Klein EA, Casey G, Rybicki BA, et al (2010) Prostate Cancer Susceptibility Variants Confer Increased Risk of Disease Progression. Cancer Epidemiology Biomarkers & Prevention 19: 2124–2132. 10.1158/1055-9965.EPI-10-0268 PMC2950095 20651075

[28] Xu J, Isaacs SD, Sun J, Li G, Wiley KE, Zhu Y, et al (2008) Association of Prostate Cancer Risk Variants with Clinicopathologic Characteristics of the Disease. Clinical Cancer Research 14: 5819–5824. 10.1158/1078-0432.CCR-08-0934 18794092 PMC2810539

[29] Eeles RA, Kote-Jarai Z, Al Olama AA, Giles GG, Guy M, Severi G, et al (2009) Identification of seven new prostate cancer susceptibility loci through a genome-wide association study. Nature Genetics 41: 1116–1121. 10.1038/ng.450 19767753 PMC2846760

[30] Eeles RA, Kote-Jarai Z, Giles GG, Olama AA, Guy M, Jugurnauth SK, et al (2008) Multiple newly identified loci associated with prostate cancer susceptibility. Nature Genetics 40: 316–321. 10.1038/ng.90 18264097

[31] Sun J, Zheng SL, Wiklund F, Isaacs SD, Li G, Wiley KE, et al (2009) Sequence variants at 22q13 are associated with prostate cancer risk. Cancer Res 69: 10–15. 10.1158/0008-5472.CAN-08-3464 19117981 PMC2705898

[32] Schumacher FR, Berndt SI, Siddiq A, Jacobs KB, Wang Z, Lindstrom S, et al (2011) Genome-wide association study identifies new prostate cancer susceptibility loci. Hum Mol Genet 20: 3867–3875. 10.1093/hmg/ddr295 21743057 PMC3168287

[33] Haiman CA, Chen GK, Blot WJ, Strom SS, Berndt SI, Kittles RA, et al (2011) Genome-wide association study of prostate cancer in men of African ancestry identifies a susceptibility locus at 17q21. Nat Genet 43: 570–573. 10.1038/ng.839 21602798 PMC3102788

[34] Kote-Jarai Z, Olama AA, Giles GG, Severi G, Schleutker J, Weischer M, et al (2011) Seven prostate cancer susceptibility loci identified by a multi-stage genome-wide association study. Nat Genet 43: 785–791. 10.1038/ng.882 21743467 PMC3396006

[35] Nam RK, Zhang WW, Trachtenberg J, Seth A, Klotz LH, Stanimirovic A, et al (2009) Utility of incorporating genetic variants for the early detection of prostate cancer. Clinical Cancer Research 15: 1787–1793. 10.1158/1078-0432.CCR-08-1593 19223501

[36] Thomas G, Jacobs KB, Yeager M, Kraft P, Wacholder S, Orr N, et al (2008) Multiple loci identified in a genome-wide association study of prostate cancer. Nature Genetics 40: 310–315. 10.1038/ng.91 18264096

[37] Ahn J, Berndt SI, Wacholder S, Kraft P, Kibel AS, Yeager M, et al (2008) Variation in KLK genes, prostate-specific antigen and risk of prostate cancer. Nature Genetics 40: 1032–1034. 10.1038/ng0908-1032 19165914 PMC3086200

[38] Wiklund F, Zheng SL, Sun J, Adami H-O, Lilja H, Hsu FC, et al (2009) Association of reported prostate cancer risk alleles with PSA levels among men without a diagnosis of prostate cancer. The Prostate 69: 419–427. 10.1002/pros.20908 19116992 PMC3348520

[39] Xu X, Valtonen-Andre C, Savblom C, Hallden C, Lilja H, Klein RJ. (2010) Polymorphisms at the Microseminoprotein-beta locus associated with physiologic variation in beta-microseminoprotein and prostate-specific antigen levels. Cancer Epidemiology, Biomarkers & Prevention 19: 2035–2042. 10.1158/1055-9965.EPI-10-0431 PMC2946372 20696662

[40] Knipe DW, Evans DM, Kemp JP, Eeles RA, Easton DF, Kote-Jarai Z et al (2014) Genetic variation in protein specific antigen detected prostate cancer and the effect of control selection on genetic association studies. Cancer Epidemiol Biomarkers & Prevention. 23(7):1356–65. 10.1158/1055-9965.EPI-13-0889 PMC4082405 24753544

[41] Jin G, Zheng SL, Lilja H, Kim ST, Tao S, Gao Z, et al (2013) Genome-wide association study identifies loci at ATF7IP and KLK2 associated with percentage of circulating free PSA. Neoplasia 15: 95–101. 23359319 10.1593/neo.121620 PMC3556942

[42] Helfand BT, Loeb S, Hu Q, Cooper PR, Roehl KA, McGuire BB, et al (2013) Personalized Prostate Specific Antigen Testing using Genetic Variants may reduce Unnecessary Prostate Biopsies. The Journal of Urology 189: 1697–1701. 10.1016/j.juro.2012.12.023 23246478 PMC3631301

[43] Loeb S, Carter HB, Walsh PC, Isaacs WB, Kettermann A, Tanaka T, et al (2009) Single Nucleotide Polymorphisms and the Likelihood of Prostate Cancer at a Given Prostate Specific Antigen Level. The Journal of Urology 182: 101–105. 10.1016/j.juro.2009.02.126 19450841 PMC4642710

[44] Johansson M, Holmström B, Hinchliffe SR, Bergh A, Stenman U-H, Hallmans G, et al (2012) Combining 33 genetic variants with prostate-specific antigen for prediction of prostate cancer: Longitudinal study. International Journal of Cancer 130: 129–137. 21328341 10.1002/ijc.25986

[45] Aly M, Wiklund F, Xu J, Isaacs WB, Eklund M, D'Amato M. (2011) Polygenic Risk Score Improves Prostate Cancer Risk Prediction: Results from the Stockholm-1 Cohort Study. European Urology 60: 21–28. 10.1016/j.eururo.2011.01.017 21295399 PMC4417350

[46] Klein RJ, Hallden C, Gupta A, Savage CJ, Dahlin A, Bjartell A, et al (2012) Evaluation of Multiple Risk—Associated Single Nucleotide Polymorphisms Versus Prostate-Specific Antigen at Baseline to Predict Prostate Cancer in Unscreened Men. European Urology 61: 471–477. 22101116 10.1016/j.eururo.2011.10.047 PMC3269546

[47] Zhang X, Mar V, Zhou W, Harrington L, Robinson MO (1999) Telomere shortening and apoptosis in telomerase-inhibited human tumor cells. Genes Dev 13: 2388–2399. 10500096 10.1101/gad.13.18.2388 PMC317024

[48] Sundin T, Hentosh P (2012) InTERTesting association between telomerase, mTOR and phytochemicals. Expert Reviews in Molecular Medicine 14: null-null. 10.1017/erm.2012.1 22455872

[49] Fletcher MNC, Castro MAA, Wang X, de Santiago I, O’Reilly M, Chin SF, et al (2013) Master regulators of FGFR2 signalling and breast cancer risk. Nat Commun 4. 10.1038/ncomms3464 PMC3778544 24043118

[50] Hindorff LA, MacArthur J, Morales J, Junkins HA, Hall PN, Klemm AK, et al A Catalog of Published Genome-Wide Association Studies. Available www.genome.gov/gwastudies.

[51] Gilbert R, Martin RM, Donovan J, Lane JA, Hamdy F, Neal DE, et al (2014) Misclassification of outcome in case—control studies: Methods for sensitivity analysis. Statistical Methods in Medical Research. 10.1177/0962280214523192 25217446

[52] Ioannidis JP, Ntzani EE, Trikalinos TA, Contopoulos-Ioannidis DG (2001) Replication validity of genetic association studies. Nat Genet 29: 306–309. 11600885 10.1038/ng749

