# Supplementary material for: Incorporating Known Genetic Variants Does Not Improve the Accuracy of PSA Testing to Identify High Risk Prostate Cancer on Biopsy
Source: PLoS One. 2015 Oct 2;10(10):e0136735. doi: 10.1371/journal.pone.0136735 (PMC4592274; doi:10.1371/journal.pone.0136735)
Supplement: S1 Fig — (DOC) [file pone.0136735.s002.doc]

**Supplementary Figure 1: Flowchart of Participants in ProtecT**

Eligible for current study = 868/2896 (30%)

PSA ≥3 & <10ng/mL, available information on SNPs, a positive biopsy result, recorded stage or Gleason score, self-identified as white.

Prostate biopsies = 7,414 (87%)

Prostate cancer = 2,896 (39%)

Eligible PSA = 8,566 (10%)

PSA ≥3 & <20ng/mL

Attended = 100,444 (44%)

PSA tested = 82,429 (82%)

Offered a PSA test= 228,966

Men aged 50-69 years
