# Supplementary material for: Incorporating Known Genetic Variants Does Not Improve the Accuracy of PSA Testing to Identify High Risk Prostate Cancer on Biopsy
Source: PLoS One. 2015 Oct 2;10(10):e0136735. doi: 10.1371/journal.pone.0136735 (PMC4592274; doi:10.1371/journal.pone.0136735)
Supplement: S1 Table — (DOC) [file pone.0136735.s003.doc]

**S1 Table: Baseline characteristics of men with prostate cancer and PSA>=3ng/mL and <10ng/mL included in the study**

|  | **High Risk** | **Low Risk** |  |
| --- | --- | --- | --- |
|  | **n** | **n** |  |
|  | **184** | **684** |  |
| **Continuous Variables** | **Mean (SD)** | **Mean (SD)** | **p for difference^a^** |
| **Age (years)** | 63.1 (4.9) | 62.4 (5.1) | 0.08 |
| **PSA (ng/mL)** | 5.4 (1.9) | 4.8 (1.6) | <0.001 |
| **BMI (kg/m**^2^) | 27.0 (3.6) | 27.2 (3.7) | 0.52 |
|  |  |  |  |
| **Categorical Variables** | **n (%)** | **n (%)** | **p for heterogeneity^b^** |
| **Agegroup** |  |  |  |
| 50-54 | 14 ( 7.61) | 72 (10.53) |  |
| 55-59 | 38 (20.65) | 154 (22.51) |  |
| 60-64 | 54 (29.35) | 212 (30.99) |  |
| >=65 | 78 (42.39) | 246 (35.96) | 0.36 |
| **Family History** |  |  |  |
| No | 164 (93.71) | 557 (91.46) |  |
| Yes | 11 ( 6.29) | 52 ( 8.54) | 0.33 |
| **Social Class** |  |  |  |
| Managerial/professional | 79 (43.41) | 337 (49.85) |  |
| Intermediate | 39 (21.43) | 98 (14.50) |  |
| Working | 64 (35.16) | 241 (35.65) | 0.06 |
| **BMI** |  |  |  |
| normal 18.5-25 | 48 (34.04) | 131 (27.12) |  |
| overweight >=25 | 64 (45.39) | 261 (54.04) |  |
| obese >=30 | 29 (20.57) | 91 (18.84) | 0.17 |
| **Weekly exercise** |  |  |  |
| None | 68 (46.90) | 222 (45.59) |  |
| 1-2 times | 47 (32.41) | 159 (32.65) |  |
| 3-4 times | 15 (10.34) | 78 (16.02) |  |
| 5+ times | 15 (10.34) | 28 ( 5.75) | 0.11 |
| **Diabetes** |  |  |  |
| No | 111 (88.80) | 422 (94.41) |  |
| Yes | 14 (11.20) | 25 ( 5.59) | 0.03 |
| **History of BPH** |  |  |  |
| No | 168 (92.31) | 610 (91.73) |  |
| Possible | 6 ( 3.30) | 31 ( 4.66) |  |
| Yes | 8 ( 4.40) | 24 ( 3.61) | 0.65 |

Men in this table have a diagnosis of prostate cancer, PSA=3-10ng/mL and had at least one available SNP result and 10 principle components for adjusting for population stratification.

Measures were collected at the time of the initial PSA test, prior to knowledge of the PSA level or diagnosis in 85% of men. Body mass index (BMI; kg/m2), represents general adiposity.

SD=standard deviation; BMI=body mass index; BPH=benign prostatic hyperplasia

^a^ Calculated using a t-test

^b^ Calculated using a Chi-squared test (χ^2^)
