# Supplementary material for: Incorporating Known Genetic Variants Does Not Improve the Accuracy of PSA Testing to Identify High Risk Prostate Cancer on Biopsy
Source: PLoS One. 2015 Oct 2;10(10):e0136735. doi: 10.1371/journal.pone.0136735 (PMC4592274; doi:10.1371/journal.pone.0136735)
Supplement: S2 Table — (DOC) [file pone.0136735.s004.doc]

**S2 Table: The association between SNPs and principal components in men with prostate cancer and PSA 3-10ng/mL**

|  |  | **rs2736098** |  |  | **rs10788160** |  |  | **rs11067228** |  |  | **rs17632542** |  |
| --- | --- | --- | --- | --- | --- | --- | --- | --- | --- | --- | --- | --- |
| **Principal Component** | **OR** | **(95% CI)** | **p-value** | **OR** | **(95% CI)** | **p-value** | **OR** | **(95% CI)** | **p-value** | **OR** | **(95% CI)** | **p-value** |
| pca1 | 0.48 | ( -1.81 , 2.78 ) | 0.68 | -1.06 | ( -3.33 , 1.22 ) | 0.36 | -0.7 | ( -3.31 , 1.90 ) | 0.6 | 0.24 | ( -0.97 , 1.46 ) | 0.69 |
| pca2 | -1.54 | ( -3.86 , 0.79 ) | 0.2 | -0.42 | ( -2.73 , 1.89 ) | 0.72 | -2.11 | ( -4.75 , 0.53 ) | 0.12 | 0.71 | ( -0.52 , 1.94 ) | 0.26 |
| pca3 | -0.01 | ( -2.36 , 2.35 ) | 1 | 0.19 | ( -2.14 , 2.53 ) | 0.87 | 1.3 | ( -1.37 , 3.97 ) | 0.34 | -0.91 | ( -2.15 , 0.33 ) | 0.15 |
| pca4 | -2.69 | ( -5.02 , -0.37 ) | 0.02 | 0.35 | ( -1.97 , 2.66 ) | 0.77 | 0.48 | ( -2.16 , 3.13 ) | 0.72 | 0.78 | ( -0.45 , 2.01 ) | 0.22 |
| pca5 | 1.83 | ( -0.50 , 4.16 ) | 0.12 | -1.13 | ( -3.44 , 1.18 ) | 0.34 | -1.11 | ( -3.75 , 1.53 ) | 0.41 | -0.28 | ( -1.52 , 0.95 ) | 0.65 |
| pca6 | 0.89 | ( -1.34 , 3.12 ) | 0.43 | -0.07 | ( -2.28 , 2.15 ) | 0.95 | -0.63 | ( -3.16 , 1.90 ) | 0.63 | 0.64 | ( -0.53 , 1.82 ) | 0.28 |
| pca7 | 0.75 | ( -1.55 , 3.05 ) | 0.52 | 0.1 | ( -2.18 , 2.38 ) | 0.93 | -1.1 | ( -3.71 , 1.50 ) | 0.41 | 0.25 | ( -0.97 , 1.46 ) | 0.69 |
| pca8 | 2.41 | ( 0.13 , 4.68 ) | 0.04 | 0.81 | ( -1.45 , 3.08 ) | 0.48 | -0.74 | ( -3.33 , 1.85 ) | 0.58 | 0.04 | ( -1.17 , 1.24 ) | 0.95 |
| pca9 | -0.8 | ( -3.05 , 1.45 ) | 0.49 | 0.18 | ( -2.05 , 2.42 ) | 0.87 | 0.19 | ( -2.36 , 2.75 ) | 0.88 | 0.2 | ( -0.99 , 1.39 ) | 0.75 |
| pca10 | -1.33 | ( -3.70 , 1.04 ) | 0.27 | 0.65 | ( -1.71 , 3.00 ) | 0.59 | 2.83 | ( 0.14 , 5.51 ) | 0.04 | -1.14 | ( -2.39 , 0.11 ) | 0.07 |

Calculated using regression, unadjusted
